# Neurological involvement in localized scleroderma: a systematic review of clinical manifestations and neuroimaging findings

**DOI:** 10.3389/fimmu.2026.1921069

**Published:** 2026-09-15

**Authors:** Yuming Chong, Wangfei Mo, Congcong Deng, Nanze Yu, Zenan Xia, Xiao Long

**Affiliations:** 1 Department of Plastic and Aesthetic Surgery, Peking Union Medical College Hospital, Chinese Academy of Medical Sciences & Peking Union Medical College, Beijing, China; 2 Chinese Academy of Medical Sciences & Peking Union Medical College, Beijing, China; 3 Department of Neurosurgery, Peking Union Medical College Hospital, Chinese Academy of Medical Sciences & Peking Union Medical College, Beijing, China

**Keywords:** localized scleroderma, Parry-Romberg syndrome (PRS), progressive facial hemiatrophy, neurological involvement, MRI, CT

## Abstract

**Background:**

Localized scleroderma (LoS) is an autoimmune disorder leading to skin sclerosis and subcutaneous tissue atrophy. Apart from the disfiguring craniofacial lesions, the extracutaneous involvement in LoS has been increasingly recognized and studied. Among all, neurological involvement is common and worth special attention.

**Objectives:**

This systematic review summarizes the neurological symptoms, neuroimaging findings, and other complementary examination results of LoS.

**Methods:**

Records were retrieved using keywords related to the disease, neurological symptoms, and neurological examinations. After excluding irrelevant literature, case reports, case series, and cohort studies were included. A statistical analysis was performed, focusing on central and peripheral nervous system symptoms, neuroimaging manifestations, and other complementary examination results.

**Results:**

A total of 242 topic-related publications reporting data on nearly 3,500 patients were finally included. The top ranked central nervous system symptoms of LoS were headache and migraine, epilepsy, and hemiparesis. Headache/migraine were more frequent in Parry-Romberg syndrome than in *en coup de sabre*, whereas epilepsy was broadly comparable across subtypes. The top peripheral nervous system symptoms were paresthesia, cranial nerve involvement, and facial pain. The top five most common manifestations on MRI were T2 white matter hyperintensity lesion, brain atrophy, abnormal gyral pattern, gliosis, and ventricular dilatation. The top three most common manifestations on CT were brain calcification, skull atrophy, and white matter hypodensity. Other complementary examinations, including electroencephalography and cerebrospinal fluid test should be selectively performed if necessary.

**Conclusion:**

Neurological involvement was common among LoS patients. Attention should be paid to the neurological manifestations of LoS at their first visit. It is recommended to perform MRI scanning for all craniofacial LoS patients, with or without neurological symptoms, at the time of diagnosis.

## Introduction

Localized scleroderma (LoS) is a rare autoimmune disorder, mainly leading to skin sclerosis and subcutaneous tissue atrophy (1, 2). A population-based study in Olmsted County, USA, from 1960 to 1993 estimated an incidence rate per 10–000 population of 0.27, with an annual increase of 3.6% (3, 4). A female predominance of 2.4 to 4.2:1 was also reported (5). LoS can occur at any age, and about one-third of cases begin in childhood (6). The mean age at onset of juvenile localized scleroderma is 6.4 to 8.7 years, and onset at birth has been reported, while the mean age at onset in adult is about 45 years (6, 7).

According to the Mayo classification, LoS can be divided into five subtypes: plaque morphea, generalized morphea, bullous morphea, linear morphea, and deep morphea (8). *En coup de sabre* (ECDS) is a subtype of linear morphea characterized by a linear lesion on the forehead or frontal scalp. The lesions on the scalp are usually accompanied by irreversible hair loss. Progressive facial hemiatrophy (PFH), also known as Parry-Romberg syndrome (PRS), frequently overlaps with ECDS (9, 10). It is a special and severe type in which lesions have extended to half of the face, leading to hemifacial atrophy of skin, subcutaneous tissue, muscle, and bone (11).

The diagnosis of LoS is often based on clinical manifestations and/or skin biopsy. Systematic and topical medications are used to control disease progression. Topical medications include glucocorticoid preparations, topical calcipotriol ointment, and tacrolimus ointment. Systematic medications include anti-inflammatory drugs and immunosuppressants. After the disease reaches a stable stage, surgical treatment such as autologous fat transplantation and skin flap transplantation can be considered to restore soft tissue volume. LoS may present as a remission-relapse course. It could recur even after many years of quiescence, especially in linear morphea of limbs (12). And juvenile patients may experience multiple relapses in adulthood (13).

In recent years, evidence from cohort studies has shown that LoS, traditionally regarded as a cutaneous autoimmune disorder, can involve a range of neurological involvement, particularly in craniofacial variants such as ECDS and PRS (14–17). Manifestations range from headaches, seizures, and cognitive or psychiatric symptoms to neuroimaging abnormalities affecting the cerebral parenchyma, ventricular system, and meninges (**Figure 1**). The mechanisms remain incompletely defined but may involve immune-mediated inflammation, vasculopathy, fibrosis, and disruption of adjacent tissues. Recognition of neurological complications is important because symptoms can precede, accompany, or follow skin disease. Multidisciplinary assessment, neuroimaging, and individualized immunomodulatory management are often required to optimize outcomes in clinical practice. This is a systematic review to comprehensively summarize the neurological symptoms and neuroimaging findings of LoS.

**Figure 1:**
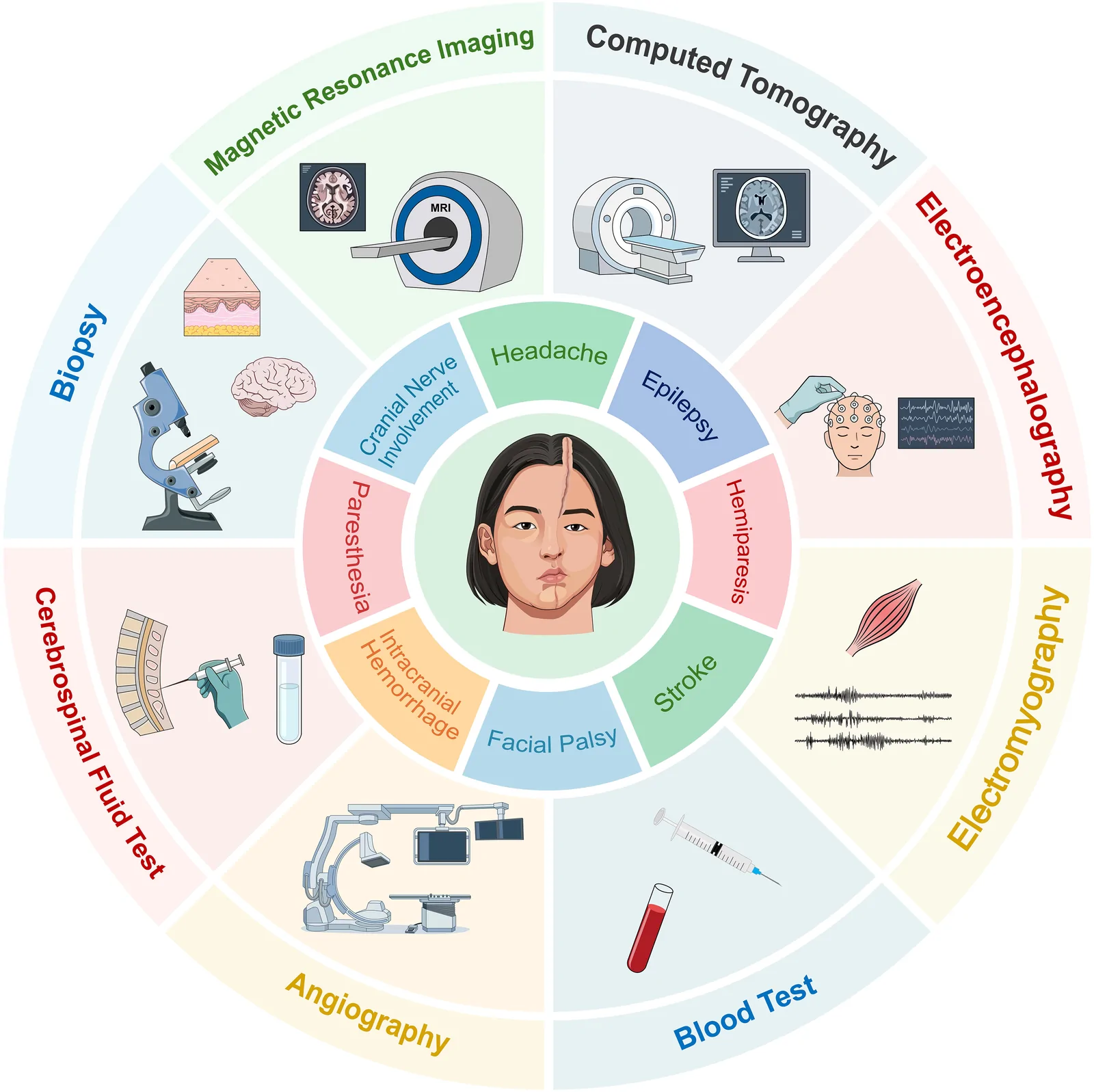
Schematic diagram of neurological involvement of localized scleroderma. SSc: systemic sclerosis.

## Methods

This systematic review was reported in accordance with the PRISMA guidelines. This review focused on LoS and its neurological involvement including clinical symptoms, neuroimaging findings, and complementary examinations. A literature search was performed in PubMed, Embase, and Cochrane Library using the following terms:

- Disease-related terms: “localized scleroderma” OR “en coup de sabre” OR “morphea” OR “Parry Romberg” OR “Parry–Romberg” OR “progressive facial hemiatrophy” OR “progressive hemifacial atrophy”
- Symptom-related terms: “brain” OR “neurological symptom*” OR “neurologic symptom*” OR “neurological involvement*” OR “neurologic involvement*” OR “neurological manifestation*” OR “neurologic manifestation*” OR “aneurysm*” OR “aneurysmal” OR “cognitive impairment*” OR “memory” OR “seizure*” OR “epilepsy” OR “headache” OR “depression” OR “anxiety” OR “mood disorder*” OR “neuropsychiatric” OR “psychosis” OR “neuropathy” OR “neuropathologic” OR “peripheral nerve*” OR “cranial nerve*” OR “carpal tunnel syndrome” OR “ulnar entrapment” OR “tarsal tunnel syndrome” OR “mononeuropathy” OR “polyneuropathy” OR “radiculopathy” OR “myelopathy” OR “autonomic nervous system” OR “nervous system”
- Complementary examination-related terms: “magnetic resonance imaging” OR “MRI” OR “magnetic resonance angiography” OR “MRA” OR “computed tomography” OR “CT” OR “electroencephalography” OR “EEG” OR “electromyography” OR “EMG” OR “neuroimaging”

The search was limited to studies published between 1947 to 2025. Only English publications were included. Manual screening was performed by two independent authors (Y.C. and W.M.) to exclude irrelevant articles and literature without detailed information for analysis. Neurological symptoms, neuroimaging manifestations, and complementary examination results were summarized. For each eligible cohort study or case series, the number of affected patients and the number assessed were extracted for main neurological symptoms and neuroimaging abnormalities. Overall analyses included all eligible cohorts, while subgroup analyses were restricted to explicitly reported ECDS, PRS, and ECDS+PRS cohort. Pooled prevalences and 95% confidence intervals were calculated using the Wilson score method. Pooled prevalences were estimated separately for each outcome and subgroup using a random-effects binomial–normal generalized linear mixed model with a logit link, fitted by maximum likelihood.

## Results

A total of 336 publications were identified for screening (**Figure 2**). After excluding non-English records, records that could not be retrieved, and irrelevant literature, 248 articles went into further analysis. Six of them were excluded because they lacked sufficient information for data extraction and quantitative analysis. Finally, this review included a total of 242 publications for review, comprising case reports, case series, and cohort studies, summing up nearly 3,500 patients (18–227).

**Figure 2:**
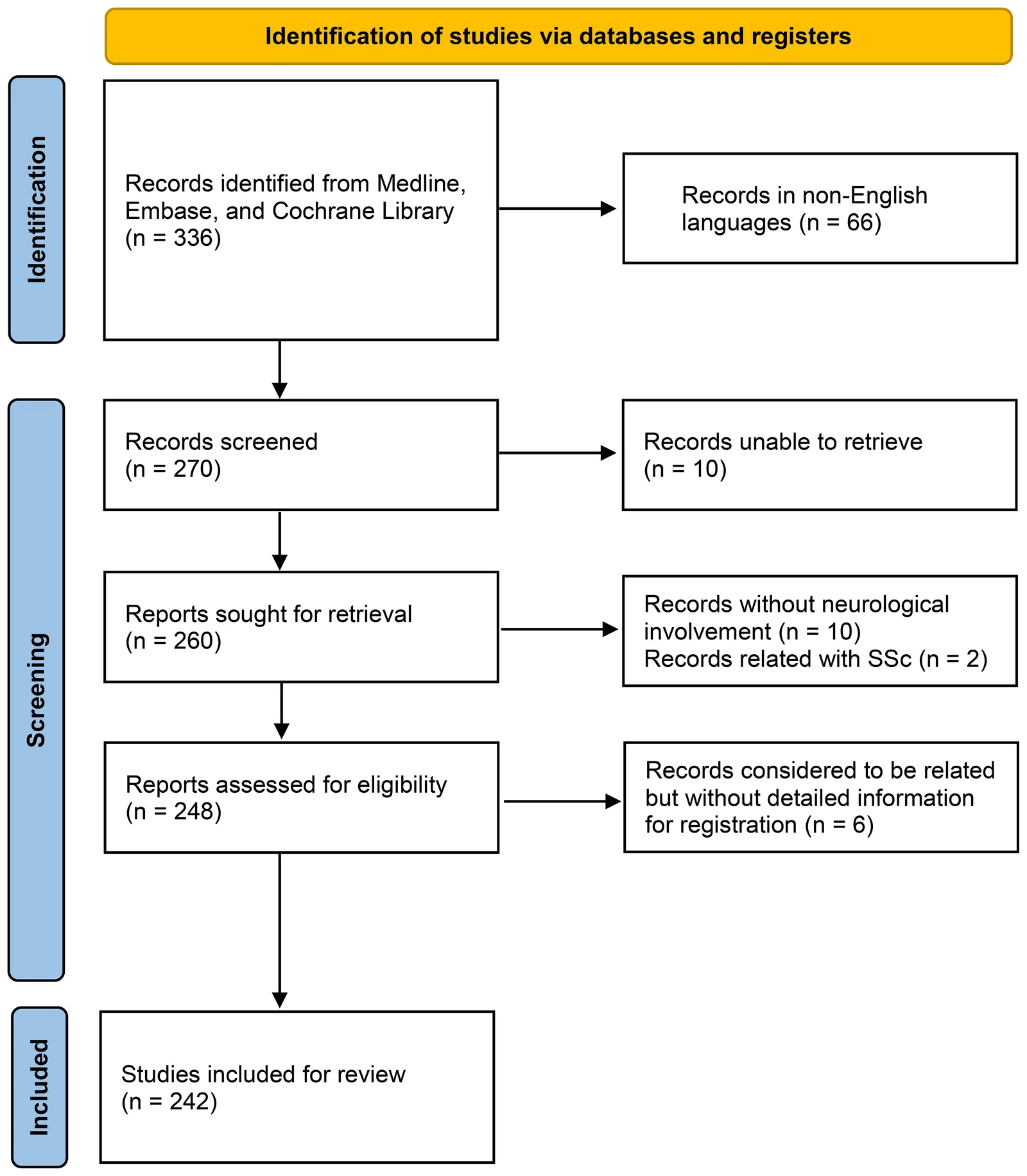
Flow diagram for this systematic review.

### Neurologic symptoms

Central nervous system (CNS) involvement is common among patients with LoS (**Table 1**). Headache/migraine was the most frequently reported CNS symptom, presenting in 384 patients, followed by epilepsy occurring in 203 cases. Other central nervous system symptoms, including hemiparesis, stroke, vomiting and nausea, and developmental retardation were also common. Other symptoms included vertigo, facial palsy, automatic activity, impaired memory, hearing loss, tinnitus, etc.

**Table 1:** Number of cases with different central nervous system symptoms in patients with localized scleroderma.

Rank	Symptom	Events	Reference
1	Headache/migraine	384	(18–78)
2	Epilepsy	203	(18, 20, 22, 26–29, 31–35, 37, 38, 40, 43, 46–49, 52–55, 57, 58, 60, 63, 65, 69, 70, 73, 76, 77, 79–157)
3	Hemiparesis	40	(32, 44, 47, 49, 60, 61, 63, 72–74, 84, 93, 101–103, 105, 108, 111, 113, 115, 116, 119, 125, 127, 129, 138, 148, 150, 153, 154, 158–165)
4	Developmental retardation	14	(48, 65, 101, 121, 129, 130, 139, 143, 148, 150, 164, 166)
5	Stroke	13	(18, 27, 33, 34, 42, 53, 72, 82, 86, 88, 167, 168)
6	Vertigo	12	(53, 71, 78, 79, 84, 88, 100, 102, 104, 148)
	Facial palsy	12	(49, 54, 58, 82, 102, 130, 131, 156, 169–171)
8	Impaired memory	8	(55, 58, 79, 97, 107, 142, 166, 167)
9	Tinnitus	7	(18, 61, 102)
10	Dysphagia	6	(41, 49, 124, 131)
	Hearing loss	6	(30, 78, 160, 162, 167, 172)
12	Impaired visuospatial function	4	(32, 52, 58, 97)
	Ataxia	4	(32, 79, 158, 173)
14	Dysarthria	3	(131, 143, 173)
15	Mirror movement	1	(109)

Sixteen cohort studies/case series (2,913 patients) contributed to the neurological-symptom meta-analysis (**Figure 3**. **Supplementary Table 1**) (18, 23, 27, 28, 32, 35, 37, 48, 49, 58, 70, 102, 126, 130, 134, 208). Headache/migraine was the most frequently reported manifestation, with a random-effects pooled prevalence of 13.2% (95% CI, 6.2%–23.9%), followed by epilepsy at 6.5% (2.7%–14.0%). Headache/migraine appeared more frequent in PRS (28.1%) and ECDS+PRS (21.1%) than in ECDS (19.2%), whereas pooled epilepsy estimates were broadly comparable across subtypes (11.7%–15.4%). Pooled prevalence data of other main neurological symptoms were shown in **Supplementary Table 1**.

**Figure 3:**
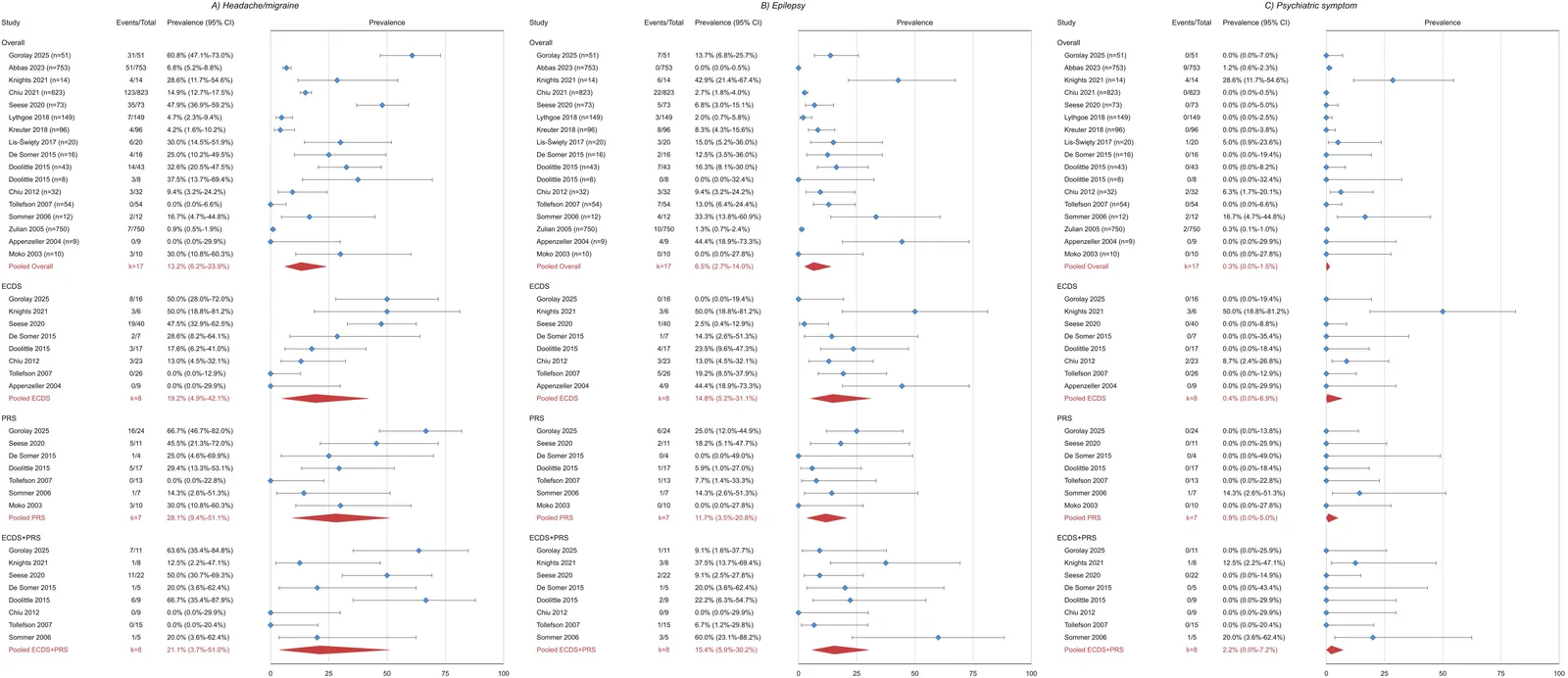
Random-effects pooled prevalence of neurological symptoms in localized scleroderma: overall and subtype-specific analysis. **(A)** Headache/migraine; **(B)** Epilepsy; **(C)** Psychiatric symptom. ECDS, *en coup de sabre*; PRS, Parry-Romberg syndrome.

Peripheral nervous system (PNS) involvement can also be observed in patients with LoS (**Table 2**). A total of 61 cases were identified with paresthesia, the most common peripheral nervous system symptom. Reported paresthesia (sensory disturbance) included unilateral paresthesia, hypoesthesia, dysesthesia, numbness, and heaviness. Cranial nerve involvement was described in 46 cases, making it the second most common symptom. Among other peripheral nervous system involvement, facial pain and peripheral neuropathy were reported in 25 and 8 cases, respectively.

**Table 2:** Number of cases with different peripheral nervous system symptoms in patients with localized scleroderma.

Rank	Symptom	Events	Reference
1	Paresthesia	61	(18, 27, 29, 34, 38, 42, 47, 50, 53–55, 58, 60, 66, 72–74, 76, 83, 93, 98, 102, 107, 132, 148, 153, 156, 158, 160, 171, 172, 174–181)
2	Cranial nerve involvement	46	
	2nd (Optic nerve)	6	(137, 156, 181–184)
	3rd (Oculomotor nerve)	7	(54, 76, 130, 131, 141, 151, 184)
	4th (Trochlear nerve)	3	(76, 79, 130)
	5th (Trigeminal nerve)	9	(49, 64, 131, 152, 172, 174, 185–187)
	6th (Abduction nerve)	8	(54, 78, 82, 128, 130, 131, 188, 189)
	7th (Facial nerve)	11	(49, 66, 69, 87, 130, 141, 169, 188, 190, 191)
	Unspecified	2	(27)
3	Facial pain	25	(18, 192–194)
4	Peripheral Neuopathy	8	(70, 102, 128, 130, 171)
5	Myalgia	7	(48, 58, 65, 155, 157, 165, 171)
6	Facial spasm	6	(49, 191, 194–196)
7	Myopathy	2	(197)
	Alien-hand syndrome	2	(52, 162)
9	Cervical sympathetic hyperactivity	1	(192)

Psychiatric symptoms can also be been observed in LoS patients (**Table 3**). Among them, anxiety (18 cases) was the most common symptom, followed by depression (16 cases), cognitive impairment (13 cases), and photophobia (13 cases). The pooled prevelence meta-analysis showed psychiatric symptoms were less common (0.3%, 0.0%–1.5%) in LoS patient population (**Figure 3**).

**Table 3:** Number of cases with psychiatric symptoms in patients with localized scleroderma.

Rank	Symptom	Events	Reference
1	Anxiety	18	(24, 27, 42, 52, 79, 84, 91, 97, 142, 198, 199)
2	Depression	16	(27, 36, 42, 52, 57, 84, 95, 97, 98, 102, 130, 199)
3	Cognitive impairment	13	(33, 52, 58, 65, 79, 84, 93, 97, 111, 125, 153, 166, 167)
	Photophobia	13	(38, 40, 53, 56, 73, 160, 179, 186, 190, 200, 201)
5	Sleep disturbance	9	(53, 91, 199)
6	Appetite change	8	(52, 56, 199)
7	Behavioral disorders	7	(52, 58, 70, 79, 139)
8	Irritability	6	(26, 53, 65, 97, 124, 139)
	Phonophobia	6	(38, 53, 73, 186, 190, 201)
10	Auditory hallucinations	5	(52, 148, 156, 157)
11	Visual hallucinations	3	(27, 52, 53)
12	Obsessive–compulsive disorder	2	(27, 198)
13	Agoraphobia	1	(99)
	Oppositional behaviour	1	(121)
	Somnolent	1	(63)
-	Unspecified	10	(23, 130)

### Neuroimaging findings

MRI and CT were the key neuroimaging methods to evaluate the neurological involvement in LoS.

MRI scans in patients with LoS reveal a diverse range of typical abnormalities (**Table 4**). T2 white matter hyperintensity was found in 174 cases, making it the most common feature on MRI. Brain parenchyma atrophy (86 cases), abnormal gyral patten (46 cases), hemorrhage (24 cases), and ventricular dilatation (24 cases) were also reported, followed by brain calcification (23 cases), gliosis (23 cases), cystic lesion (19 cases), blurring white-gray matter (16 cases), aneurysm & vascular malformation (14 cases), cerebral infarction & ischemia (13 cases), grey matter abnormality (12 cases), and leptomeningeal enhancement (11 cases).

**Table 4:** Number of cases with abnormalities on brain MRI in patients with localized scleroderma.

Rank	Manifestation	Events	Reference
1	T2 white matter hyperintensity	174	(20, 22, 26, 27, 29–34, 37, 38, 40–43, 47–50, 52–55, 58–60, 66, 67, 69, 71–74, 79–89, 91–94, 97, 98, 101–103, 105, 107, 110, 111, 113, 116–118, 120–123, 126, 129–134, 138, 142–144, 146, 148, 153, 154, 158, 159, 161–163, 165, 172–175, 180, 183, 189, 194, 201–211)
2	Brain atrophy	86	(18, 24, 26, 27, 31, 32, 34, 41, 43, 48, 49, 52, 55, 65, 66, 82–84, 88, 98, 101–104, 107, 108, 111, 115, 117–120, 125, 127, 129, 131, 134, 138, 139, 143, 149, 152, 161, 162, 164, 166, 173, 174, 193, 205, 208, 212–217)
3	Abnormal gyral pattern	46	(24, 27, 29, 31, 32, 40, 47, 49, 50, 54, 58, 60, 73, 74, 83, 97, 111, 129, 131, 132, 134, 142, 146, 148, 153, 166, 174, 205)
4	Hemorrhage	24	(18, 20, 22, 34, 38, 49, 56, 60, 63, 72, 81, 87, 92, 93, 100, 103, 106, 172, 174, 204)
	Ventricular dilatation	24	(26, 29, 31, 73, 82–84, 97, 102, 107, 111, 119, 130–132, 138, 139, 148, 153, 166, 174, 205, 207)
6	Calcification	23	(20, 27, 31, 34, 39, 40, 42, 47, 58, 60, 102, 103, 105, 130, 134, 149, 211, 218)
	Gliosis	23	(31, 34, 37, 84, 88, 101, 104, 111, 120, 137, 148, 162, 175, 182, 183, 219)
8	Cystic lesion	19	(18, 26, 31, 38, 44, 55, 83, 87, 137, 148, 175, 207)
9	Blurring white-gray matter	16	(74, 83, 130, 132, 134, 148)
10	Aneurysm & vascular malformation	14	(59, 61, 63, 68, 70, 84, 104, 147, 179, 180, 189, 220)
11	Cerebral infarction & ischemia	13	(56, 63, 72, 82, 129, 159, 165, 167, 168, 174, 180, 186, 212)
12	Grey matter abnormality	12	(32, 49, 112)
13	Leptomeningeal enhancement	11	(27, 32, 47, 50, 73, 74, 146, 204)
14	Edema	10	(31, 44, 47, 52, 54, 87, 105, 112)
15	Encephalomalacia	9	(38, 49, 82, 87, 102, 182, 183)
16	Cortex thickening	8	(31, 50, 52, 54, 74, 83, 148)
17	Cavernoma	7	(27, 60, 80, 92, 175, 218)
	Vasculitis	7	(56, 59, 70, 94, 102, 126)
19	Demyelination	4	(55, 102, 211)
	Lateral ventricle asymmetry	4	(49, 221)
	Tumor	4	(47, 126, 210, 222)
22	Agenesis of corpus callosum	3	(97, 108, 154)
23	Porencephaly	2	(27, 150)
	Subdural effusion	2	(131, 135)
25	Cavum septum pellucidum	1	(198)

Eleven MRI cohorts (411 patients) were included in the neuroimaging meta-analysis (**Figure 4**, **Supplementary Table 2**) (18, 27, 32, 37, 48, 49, 58, 102, 130, 134, 208). T2 white-matter hyperintensities were the most prevalent MRI abnormality overall (15.0%, 7.0%–27.3%) and were most frequent in the ECDS+PRS subgroup (27.1%, 8.2%–45.2%). Brain atrophy was less common overall (4.0%, 0.5%–19.4%), while abnormal gyral patterns were rare and imprecisely estimated (0.9%, 0.0%–12.3%). Pooled prevalence data of other main neuroimaging abnormalities were shown in **Supplementary Table 2**.

**Figure 4:**
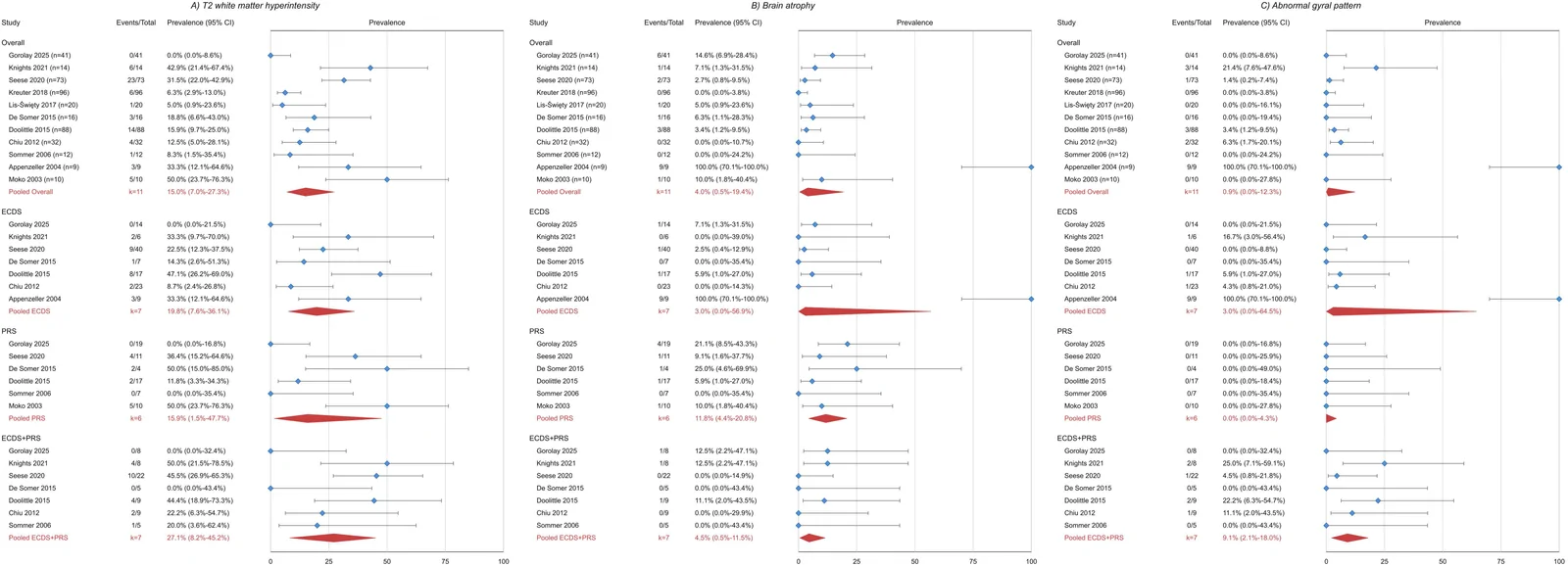
Random-effects pooled prevalence of magnetic resonance imaging abnormalities in localized scleroderma: overall and subtype-specific analysis. **(A)** T2 white matter hyperintensity; **(B)** Brain atrophy; **(C)** Abnormal gyral pattern. ECDS, *en coup de sabre*; PRS, Parry-Romberg syndrome.

CT scans may also indicate underlying abnormalities (**Table 5**). Brain calcification (39 cases), skull atrophy (21 cases), and white matter hypodensity lesion (16 cases) were findings with high frequency. Other findings on CT included gliosis (7 cases), cerebral nodular lesion (6 cases), abnormal gyral pattern (6 cases), cerebral cortex atrophy (5 cases), enophthalmos (5 cases), hemorrhage (5 cases), etc.

**Table 5:** Number of cases with abnormalities on head CT in patients with localized scleroderma.

Rank	Manifestation	Events	Reference
1	Brain calcification	39	(18, 31, 34, 38, 40, 42, 43, 47–49, 54, 63, 65, 69, 74, 76, 77, 83, 85, 96, 104, 105, 112, 121, 134, 140, 144, 145, 147, 148, 150, 156, 163, 189, 209, 211, 222)
2	Skull atrophy	21	(24, 29, 57, 58, 68, 76, 83, 93, 99, 134, 141, 163, 204, 217, 223, 224)
3	White matter hypodensity	16	(26, 31, 32, 34, 38, 52, 74, 83, 85, 96, 98, 126, 144, 165, 206)
4	Gliosis	7	(37)
5	Cerebral nodular lesion/mass	6	(29, 48, 63, 92, 99, 222)
	Abnormal gyral pattern	6	(24, 32, 47, 50, 132, 205)
6	Cerebral cortex atrophy	5	(29, 43, 76, 152, 217)
	Enophthalmos	5	(114, 147, 225–227)
	Hemorrhage	5	(56, 60, 61, 92, 106)
10	Aneurysm	4	(63, 68, 76, 220)
	Ventricular dilatation	4	(63, 76, 148, 156)
12	Edema	3	(29, 60, 92)
	White matter attenuation change	3	(42, 207, 211)
14	Encephalomalacia	2	(69, 74)
	Porencephaly	2	(150, 156)
16	Cavum septum pellucidum	1	(198)
	Hydrocephalus	1	(63)
	Hypoplastic left carotid canal	1	(119)

### Complementary examinations

Electroencephalography (EEG) was the most frequently applied complementary examination, especially in patients with seizures (**Table 6**). EEG was abnormal in 100 of the 151 patients (66%) who underwent the examination. Angiography was the second most commonly used examination; it was performed in 114 patients and yielded positive findings in 44 (38%). Skin biopsy was performed in 82 cases, most of which showed positive results (93%). Skin biopsy specimens usually showed swelling of connective tissues, excessive and thickened collagen fibers, perivascular infiltration of lymphocytes and plasma cells, proliferated adnexal glands, and atrophy of the subcutaneous fat. Brain biopsy was performed in 29 cases, all of which reported abnormal results, including perivascular inflammatory infiltrates, gliosis, fibrosis of leptomeninges, astrocytosis, focal cerebral necrosis, focal vessel obliteration, vessels proliferation, and cortical disarray. In addition, other complementary examinations included cerebral spinal fluid (CSF) test (67 cases, 51% positive rate), electromyography (37 cases, 73% positive rate), muscle biopsy (6 cases, 100% positive rate), and orbital biopsy (1 case, 100% positive rate).

**Table 6:** Other complementary examination results of patients with localized scleroderma.

Complementary examination	Events	Percentage
Electroencephalography	151	
Normal	51	34%
Abnormal	100	66%
Cerebral spinal fluid	67	
Normal	33	49%
Abnormal	34	51%
Angiography	114	
Normal	70	61%
Abnormal	44	39%
Skin biopsy	82	
Normal	6	7%
Abnormal	76	93%
Brain tissue biopsy	29	
Normal	0	0
Abnormal	29	100%
Muscle biopsy	6	
Normal	0	0
Abnormal	6	100%
Orbital biopsy	1	
Normal	0	0
Abnormal	1	100%
Electromyography	37	
Normal	10	27%
Abnormal	27	73%

## Discussion

Neurological involvement in LoS, particularly in the linear craniofacial subtype, represents one of the most clinically significant extracutaneous manifestations of this disease. Historically regarded as a condition confined to the skin and subcutaneous tissues, accumulating evidence from case reports, case series, and cohort studies has substantially revised this understanding, revealing a spectrum of central nervous system, peripheral nervous system, and psychiatric complications that may precede, accompany, or follow skin involvement.

Among CNS manifestations identified in this review, headache and migraine emerged as the most frequently reported neurological symptoms, consistent with prior literature. Their occurrence is unlikely to represent incidental comorbidity; rather, it may reflect identifiable structural and vascular pathology. Leptomeningeal inflammation, evidenced by contrast-enhanced MRI in a substantial proportion of patients, can directly irritate pain-sensitive meningeal afferents and generate headache phenotypes indistinguishable from primary migraine. This mechanism is supported by pediatric neuroimaging series demonstrating persistent leptomeningeal enhancement ipsilateral to the cutaneous lesion, often co-occurring with adjacent parenchymal T2 signal abnormality and progressive white matter involvement (27). Additionally, focal cortical pathology—including peri-cystic lesion gliosis and abnormal gyral patterning—may lower the threshold for cortical spreading depression, a well-established electrophysiological substrate of migraine, thereby coupling structural lesions to recurrent migraine. Epilepsy constitutes one of the more severe CNS manifestations and has been documented in a substantial proportion of patients with LoS. The structural changes that trigger epilepsy in LoS patients are heterogeneous: T2 white matter hyperintensity lesions in periventricular and subcortical locations may disrupt cortico-cortical inhibitory networks; focal cortical abnormalities, including abnormal gyral patterns and peri-cystic lesion gliosis, represent intrinsically epileptogenic focus; and microhemorrhages, almost confined to the hemisphere ipsilateral to the skin lesion, may serve as chronic epileptogenic trigger through iron and hemoglobin deposition (17, 228). Critically, neuropathological evidence from brain biopsy has demonstrated small vessel lymphocytic vasculitis, with co-existing acute and chronic cortical ischemia directly attributable to immune-mediated endothelial injury, linking the inflammatory process mechanistically to seizure generation (97). The severity of ipsilateral intracranial abnormalities on MRI has been identified as a significant risk factor for the development of epilepsy in PRS patients, underscoring the clinical value of early neuroimaging in this population (229). Hemiparesis, though reported with lower frequency, carries substantial functional implications. Its structural basis possibly involves ischemic injury to the corticospinal tract. In neuroimaging series of ECDS and PRS, hemiparesis has been documented in association with ipsilateral hemispheric abnormalities including white matter disease, cortical atrophy, and evidence of prior ischemic injury, consistent with a slowly evolving inflammatory process rather than acute atherosclerotic stroke (49). Stroke-like episodes have been documented in patients with LoS. Case reports have described acute ischemic infarction at sites including the posterior limb of the internal capsule, with proposed mechanisms encompassing ipsilateral cerebrovascular abnormality, migraine-associated vasospasm, and patent foramen ovale as potential risk factors (159). A proinflammatory state associated with ECDS/PRS may further predispose affected patients to small-vessel vasculopathy, though the precise stroke mechanism in individual cases frequently remains undetermined (168).

Developmental retardation in pediatric patients deserves particular attention. Case reports have documented mental and motor regression occurring in young children with ECDS, with MRI demonstrating hippocampal atrophy ipsilateral to the skin lesion as a structural correlation; critically, neurological symptoms may antedate the appearance of visible skin changes by over a year, creating a substantial risk of diagnostic delay (65). Beyond direct structural injury, refractory epilepsy—which develops in approximately 40% of pediatric PRS patients with seizures—carries a significant independent impact on neurodevelopment, compounding the cognitive and motor burden in affected children (228).

Psychiatric symptoms, particularly depression and anxiety, constitute a distinct and clinically important dimension of neurological involvement in LoS. Their pathogenesis is likely multifactorial: direct involvement of limbic and prefrontal circuitry by periventricular or subcortical white matter lesions may disrupt emotion regulation which relies on a delicate interplay between large-scale networks, while chronic pain, disfigurement, and the psychosocial burden of a visible facial lesion independently predispose patients to affective disorders (126). In a small pediatric ECDS/PRS MRI series, psychiatric diagnoses occurred both in patients with and without MRI abnormalities and showed no association with abnormal neuroimaging, although statistical power was limited (27). Consistently, a systematic review of 690 children with juvenile localized scleroderma found qualitative evidence of reduced self-worth, stigma, teasing, and treatment burden, while emphasizing the lack of robust prevalence data for formal depressive or anxiety disorders (230). Therefore, psychiatric symptoms should not be considered surrogate markers of CNS inflammation; routine psychological screening, timely mental-health referral, and parallel neurological assessment are warranted, particularly when affective symptoms co-occur with headache, seizures, or cognitive deficits.

The involvement of PNS, though less extensively characterized than CNS disease, was consistently identified across case reports and small-scale cohort studies. In a systematic review of neurological involvement in LoS, cranial nerve involvement and peripheral sensorimotor neuropathy were among the documented PNS manifestations, though their precise prevalence in LoS specifically remains difficult to establish due to the predominance of CNS-focused reports (231). Paresthesia was reported to be the most frequently reported PNS symptom, followed by cranial nerve involvement and facial pain. Paresthesia may reflect demyelinating or axonal peripheral neuropathy arising from perivascular inflammation or epineural fibrosis. Cranial nerve involvement, most commonly affecting trigeminal and facial nerves, may result from direct perineural infiltration by fibrotic tissue or vascular injury to the vasa nervorum (172). The lack of nerve conduction studies and histological evidence leaves the pathophysiological basis of PNS involvement incompletely understood, and more studies are expected to decode the mechanism behind the symptoms.

Management of the neurological symptoms requires continuous medication with close follow-up. Seizures are frequently reported and can represent a severe neurological manifestation. Treatment strategies were individualized and included antiepileptic drugs (levetiracetam, carbamazepine, oxcarbazepine, lamotrigine, clobazam), corticosteroids (methylprednisolone), immunosuppressants (methotrexate, mycophenolate mofetil), and symptomatic therapies. Specific agents were selected based on seizure type and tolerability. Among seizure patients, most achieved symptom-free status or improvement with combination therapy, though some cases showed poor response or deterioration. Corticosteroids appeared effective in reducing inflammatory lesions, but relapse could occur upon discontinuation. Migraine is the most common neurological symptom. Acute NSAIDs or paracetamol and standard prophylaxis with propranolol, amitriptyline, flunarizine, topiramate, or valproate may be used, although reported responses were heterogeneous and simple analgesics were frequently insufficient (84). For new or relapsing hemiparesis, in a five-patient neurological series, corticosteroid induction followed by methotrexate or azathioprine yielded clinical improvement in three patients and radiological improvement in two; notably, one patient with recurrent transient hemiparesis remained asymptomatic for four years after methotrexate was introduced (47). For facial spasm, carbamazepine or clonazepam often provides incomplete relief, whereas botulinum toxin injections are effective in most reported cases; microvascular decompression should be reserved for demonstrated vascular compression (232). Overall, treatment response was heterogeneous, underscoring the need for multidisciplinary, individualized management approaches for neurologic manifestations in LoS.

Neuroimaging findings on MRI are diverse. T2 white matter hyperintensity represented the most prevalent MRI abnormality, reported across multiple independent series. It may reflect perivascular inflammation, focal demyelination, or ischemic change—the precise histopathological correlates of which remain an area of future investigation (65). Brain atrophy, frequently ipsilateral to the cutaneous lesion in craniofacial cases, reflects the progressive nature of the underlying process. Abnormal gyral patterns have been reported in a subset of cases and may indicate disruption of cortical development associated with early-onset disease. Gliosis, identified as T2/FLAIR signal abnormality in affected regions, represents a consistent marker of prior inflammatory or ischemic injury. Focal cortical lesions, leptomeningeal enhancement, and vascular abnormalities have been reported with lower but clinically important frequency. Notably, MRI abnormalities have been identified in neurologically asymptomatic patients, suggesting all patients with LoS, with or without neurological symptoms, are recommended to receive brain MRI scanning at the time of diagnosis (70).

Ancillary investigations should be guided by the clinical phenotype and MRI findings. EEG should be used selectively in patients with suspected or confirmed seizures. Of the reported EEGs, 80% were abnormal, and 65% showed abnormalities localized to the cerebral hemisphere ipsilateral to the cutaneous lesion (88). CSF analysis is not a screening test, but may assist in encephalopathy, progressive focal/cognitive decline, or enhancing/multifocal lesions by excluding infection, demyelination, and malignancy. Abnormal inflammatory CSF profiles support, but do not specifically establish, intrathecal inflammation. Vascular imaging is appropriate for stroke-like or hemorrhagic presentations. Brain biopsy should be reserved for progressive or mass-like lesions when vasculitis, neoplasia, infection, or demyelination remain unresolved and tissue findings would change management (97).

The mechanisms underlying neurological involvement in craniofacial linear localized scleroderma remain incompletely understood and are likely multifactorial. An immune-mediated and vasculopathic process is the leading explanation. Localized scleroderma is associated with autoantibodies, lymphocytic and macrophage infiltration, and a cytokine profile in which early Th1/Th17-associated inflammation may transition to Th2/TGF-β-driven fibrosis (7, 233, 234). Intracranial biopsy reports in ECDS have demonstrated leptomeningeal and small-vessel sclerosis, lymphocytic inflammation, and vasculitic changes (143, 149). Thus, immune-mediated microvasculopathy, with secondary ischemia, demyelination, gliosis, or cortical injury, may represent the proximate substrate for seizures, headache, and focal neurological deficits. Secondly, the trigeminal-neurogenic hypothesis is supported by the distribution of lesions in trigeminal sensory territories, together with the frequent ipsilateral relationship of cutaneous disease, MRI abnormalities, and epileptogenic foci (88, 235). However, direct evidence of inflammatory propagation along the trigeminal nerve is lacking; this hypothesis is therefore best regarded as an anatomical model rather than an established mechanism. Furthermore, a developmental mosaic-susceptibility hypothesis has also been proposed. The close conformity of linear morphoea to Blaschko lines is compatible with postzygotic mosaicism, whereby a clonally distributed population of developmentally altered cells may be selectively vulnerable to a subsequent inflammatory trigger (236). Nevertheless, in a paired-biopsy next-generation sequencing study of linear morphea, no recurrent disease-associated gene or somatic single-nucleotide variant was identified, arguing against primary epidermal mosaicism (237).

Several limitations should be acknowledged. First, the included studies differed substantially in their clinical descriptions, terminology, and level of detail. Minor or secondary symptoms may therefore have been underreported or missed in the pooled analysis. Second, topic-related publications might originate from the same center, resulting in overlapping cohorts or repeated reporting of individual cases. Such overlap could lead to an overrepresentation of selected manifestations or more severe presentations. Third, only English-language publications were included. This criterion may have introduced language bias and contributed to an uneven geographic and ethnic distribution of the available evidence. Accordingly, the reported frequencies should be interpreted as estimates derived from the published English-language literature rather than as definitive population-based prevalence figures. Universal extrapolation of these prevalence estimates should therefore be avoided.

## Conclusion

It is not uncommon for LoS patients to have neurological involvement. This systematic review summarizes the neurological symptoms and neuroimaging abnormalities acssociated with LoS. Attention should be paid to the neurological manifestations of LoS at their first visit, particularly common symptoms such as headache, migraine, epilepsy, and hemiparesis. Across subtypes, headache/migraine were more frequent in PRS and ECDS+PRS than in ECDS, whereas epilepsy was broadly comparable across subtypes. In addition, a variety of abnormalities can be observed on MRI and CT. Thus, brain MRI at diagnosis should be considered for patients with craniofacial LoS, regardless of whether neurological symptoms are present. For patients with severe neurological manifestations, MRI follow-up should be considered.

## Data Availability

The original contributions presented in the study are included in the article/**Supplementary Material**. Further inquiries can be directed to the corresponding authors.

## Supplementary material

Supplementary Table 1 Number of cases, percentage, and pooled prevalence of neurological symptoms in localized scleroderma: overall and subtype-specific analysis. ECDS, *en coup de sabre*; PRS, Parry-Romberg syndrome.

Supplementary Table 2 Number of cases, percentage, and pooled prevalence of neuroimaging abonormalities in localized scleroderma: overall and subtype-specific analysis. ECDS, *en coup de sabre*; PRS, Parry-Romberg syndrome.
