# Supplementary material for: Neurological involvement in localized scleroderma: a systematic review of clinical manifestations and neuroimaging findings
Source: Front Immunol. 2026 Sep 15;17:1921069. doi: 10.3389/fimmu.2026.1921069 (PMC13619469; doi:10.3389/fimmu.2026.1921069)
Supplement: Supplementary file 1 [file Table1.docx]

**Supplementary table 1. Number of cases, percentage, and pooled prevalence of neurological symptoms in localized scleroderma: overall and subtype-specific analysis**

|  |  | **Headache/ migraine** | **Epilepsy** | **Hemiparesis** | **Stroke** | **Developmental retardation** | **Vertigo** | **Facial palsy** | **Impaired memory** | **Tinnitus** | **Psychiatric symptom** |
| --- | --- | --- | --- | --- | --- | --- | --- | --- | --- | --- | --- |
| Gorolay 2025 (n=51) | ECDS (n=16) | 8 (50%) |  |  |  |  |  |  |  | 1 (6.3%) |  |
|  | PRS (n=24) | 16 (66.7%) | 6 (25%) |  | 1 (4.2%) |  |  |  |  | 2 (8.3%) |  |
|  | ECDS+PRS (n=11) | 7 (63.6%) | 1 (9.1%) |  | 1 (9.1%) |  |  |  |  | 2 (18.2%) |  |
| Abbas 2023 (n=753) | Not specified | 51 (6.8%) |  |  |  |  |  |  |  |  | 9 (1.2%) |
| Knights 2021 (n=14) | ECDS (n=6) | 3 (50%) | 3 (50%) |  | 1 (16.7%) |  |  |  |  |  | 3 (50%) |
|  | ECDS+PRS (n=8) | 1 (12.5%) | 3 (37.5%) |  |  |  |  |  |  |  | 1 (12.5%) |
| Chiu 2021 (n=823) | Linear (n=462) | 101 (21.86%) | 20 (4.3%) |  |  |  |  |  |  |  |  |
|  | Plaque (n=235) | 12 (5.1%) | 2 (0.9%) |  |  |  |  |  |  |  |  |
|  | Generalized (n=66) | 7 (10.6%) |  |  |  |  |  |  |  |  |  |
|  | Mixed (n=60) | 3 (5%) |  |  |  |  |  |  |  |  |  |
| Seese 2020 (n=73) | ECDS (n=40) | 19 (47.5%) | 1 (2.5%) |  |  |  |  |  |  |  |  |
|  | PRS (n=11) | 5 (45.5%) | 2 (18.2%) |  |  |  |  |  |  |  |  |
|  | ECDS+PRS (n=22) | 11 (50%) | 2 (9.1%) |  |  |  |  |  |  |  |  |
| Lythgoe 2018 (n=149) | Not specified | 7 (4.7%) | 3 (2%) |  |  |  |  |  |  |  |  |
| Kreuter 2018 (n=96) | Not specified | 4 (4.2%) | 8 (8.3%) |  |  |  |  |  |  |  |  |
| Lis-Święty 2017 (n=20) | Not specified | 6 (30%) | 3 (15%) | 1 (5%) |  |  | 2 (10%) | 1 (5%) |  | 1 (5%) | 1 (5%) |
| De Somer 2015 (n=16) | ECDS (n=7) | 2 (28.6%) | 1 (14.3%) |  |  | 1 (14.3%) |  |  |  |  |  |
|  | PRS (n=4) | 1 (25%) |  |  |  | 1 (25%) |  |  |  |  |  |
|  | ECDS+PRS (n=5) | 1 (20%) | 1 (20%) |  |  |  |  |  |  |  |  |
| Doolittle 2015 (n=43) | ECDS (n=17) | 3 (17.6%) | 4 (23.5%) |  |  |  |  |  |  |  |  |
|  | PRS (n=17) | 5 (29.4%) | 1 (5.9%) |  |  |  |  |  |  |  |  |
|  | ECDS+PRS (n=9) | 6 (66.7%) | 2 (22.2%) | 1 (11.1%) |  |  |  | 1 (11.1%) |  |  |  |
| Doolittle 2015 (n=8) | Not specified | 3 (37.5%) |  |  |  |  |  | 1 (12.5%) |  |  |  |
| Chiu 2012 (n=32) | ECDS (n=23) | 3 (13%) | 3 (13%) |  |  |  |  | 1 (4.3%) | 1 (4.3%) |  | 2 (8.7%) |
|  | ECDS+PRS (n=9) |  |  |  |  |  |  |  |  |  |  |
| Tollefson 2007 (n=54) | ECDS (n=26) |  | 5 (19.2%) |  |  |  |  |  |  |  |  |
|  | PRS (n=13) |  | 1 (7.7%) |  |  |  |  |  |  |  |  |
|  | ECDS+PRS (n=15) |  | 1 (6.7%) |  |  |  |  |  |  |  |  |
| Sommer 2006 (n=12) | PRS (n=7) | 1 (14.3%) | 1 (14.3%) |  |  | 1 (14.3%) |  |  |  |  | 1 (14.3%) |
|  | ECDS+PRS (n=5) | 1 (20%) | 3 (60%) |  |  |  |  | 1 (20%) |  |  | 1 (20%) |
| Zulian 2005 (n=750) | Not specified | 7 (0.9%) | 10 (1.3%) |  |  |  |  |  |  |  | 2 (0.3%) |
| Appenzeller 2004 (n=9) | ECDS |  | 4 (44.4%) |  |  |  |  |  |  |  |  |
| Moko 2003 (n=10) | PRS | 3 (30%) |  |  |  |  |  |  |  |  |  |
| **Events (%)** | All subtypes (n=2913) | 297 (10.2%) | 91 (3.1%) | 2 (0.1%) | 3 (0.1%) | 3 (0.1%) | 2 (0.1%) | 5 (0.2%) | 1 (0.0%) | 6 (0.2%) | 20 (0.7%) |
|  | ECDS (n=144) | 38 (26.4%) | 21 (14.6%) | 0 | 1 (0.7%) | 1 (0.7%) | 0 | 1 (0.7%) | 1 (0.7%) | 1 (0.7%) | 5 (3.5%) |
|  | PRS (n=86) | 31 (36%) | 11 (12.8%) | 0 | 1 (1.2%) | 2 (2.3%) | 0 | 0 | 0 | 2 (2.3%) | 1 (1.2%) |
|  | ECDS+PRS (n=84) | 27 (32.1%) | 13 (15.5%) | 1 (1.2%) | 1 (1.2%) | 0 | 0 | 2 (2.4%) | 0 | 2 (2.4%) | 2 (2.4%) |
| **Pooled prevalence %**  **(95% CI)** | All subtypes | 13.16 (6.18–23.95) | 6.52 (2.67–14.03) | 0.015 (<0.001–0.222) | 0.007 (<0.001–0.219) | 0.002 (<0.001–0.176) | <0.001 (<0.001–0.082) | 0.113 (0.001–0.676) | 0.003 (<0.001–0.132) | 0.004 (<0.001–0.197) | 0.311 (0.011–1.498) |
|  | ECDS | 19.151 (4.891–42.069) | 14.790 (5.228–31.146) | 0.000 (0.000–2.059) | 0.196 (<0.001–2.910) | 0.262 (<0.001–2.952) | 0.000 (0.000–2.059) | 0.694 (<0.001–3.022) | 0.694 (<0.001–3.022) | 0.694 (<0.001–3.022) | 0.396 (<0.001–6.871) |
|  | PRS | 28.054 (9.388–51.084) | 11.741 (3.545–20.776) | 0.000 (0.000–3.423) | 1.163 (<0.001–5.020) | 1.591 (<0.001–7.731) | 0.000 (0.000–3.423) | 0.000 (0.000–3.423) | 0.000 (0.000–3.423) | 1.585 (<0.001–6.910) | 0.862 (<0.001–5.010) |
|  | ECDS+PRS | 21.119 (3.709–50.958) | 15.389 (5.880–30.198) | 1.190 (<0.001–5.137) | 1.190 (<0.001–5.137) | 0.000 (0.000–3.504) | 0.000 (0.000–3.504) | 2.343 (0.004–7.174) | 0.000 (0.000–3.504) | 0.356 (<0.001–5.853) | 2.154 (0.003–7.184) |

ECDS: *en coup de sabre*, PRS: Parry-Romberg syndrome.

**Supplementary table 2. Number of cases, percentage, and pooled prevalence of neuroimaging abonormalities in localized scleroderma: overall and subtype-specific analysis.**

|  |  | **T2 white matter hyperintensity** | **Brain**  **atrophy** | **Abnormal  gyral pattern** | **Gliosis** | **Ventricular  dilatation** | **Calcification** | **Hemo-**  **rrhage** | **Cystic lesion** | **Leptomeningeal enhancement** | **Encepha-**  **lomalacia** |
| --- | --- | --- | --- | --- | --- | --- | --- | --- | --- | --- | --- |
| Gorolay 2025 (n=41) | ECDS (n=14) |  | 1 (7.1%) |  |  |  |  |  | 1 (7.1%) |  |  |
|  | PRS (n=19) |  | 4 (21.1%) |  |  |  |  | 3 (15.8%) | 3 (15.8%) |  |  |
|  | ECDS & PRS (n=8) |  | 1 (12.5%) |  |  |  |  | 2 (25%) | 1 (12.5%) |  |  |
| Knights 2021 (n=14) | ECDS (n=6) | 2 (33.3%) |  | 1 (16.7%) |  |  | 2 (33.3%) |  |  | 2 (33.3%) |  |
|  | ECDS &PRS (n=8) | 4 (50%) | 1 (12.5%) | 2 (25%) |  |  | 3 (37.5%) |  |  | 2 (25%) |  |
| Seese 2020 (n=73) | ECDS (n=40) | 9 (22.5%) | 1 (2.5%) |  |  |  |  |  |  |  |  |
|  | PRS (n=11) | 4 (36.4%) | 1 (9.1%) |  |  |  |  |  |  |  |  |
|  | ECDS & PRS (n=22) | 10 (45.5%) |  | 1 (4.5%) |  |  |  |  |  | 1 (4.5%) |  |
| Kreuter 2018 (n=96) | Not specified | 6 (6.3%) |  |  | 7 (7.3%) |  |  |  |  |  |  |
| Lis-Święty 2017 (n=20) | Not specified | 1 (5%) | 1 (5%) |  |  | 1 (5%) | 1 (5%) |  |  |  | 1 (5%) |
| De Somer 2015 (n=16) | ECDS (n=7) | 1 (14.3%) |  |  |  |  |  |  |  |  |  |
|  | PRS (n=4) | 2 (50%) | 1 (25%) |  |  |  |  |  |  |  |  |
|  | ECDS+PRS (n=5) |  |  |  |  |  |  |  |  |  |  |
| Doolittle 2015 (n=88) | ECDS (n=17) | 8 (47.1%) | 1 (5.9%) | 1 (5.9%) |  |  |  | 1 (5.9%) |  |  | 3 (17.6%) |
|  | PRS (n=17) | 2 (11.8%) | 1 (5.9%) |  |  |  |  |  |  |  |  |
|  | ECDS+PRS (n=9) | 4 (44.4%) | 1 (11.1%) | 2 (22.2%) |  |  |  |  |  |  |  |
| Chiu 2012 (n=32) | ECDS (n=23) | 2 (8.7%) |  | 1 (4.3%) |  |  |  |  |  |  |  |
|  | ECDS+PRS (n=9) | 2 (22.2%) |  | 1 (11.1%) |  |  | 1 (11.1%) |  |  |  |  |
| Sommer 2006 (n=12) | PRS (n=7) |  |  |  |  |  |  |  |  |  |  |
|  | ECDS+PRS (n=5) | 1 (20%) |  |  |  | 1 (20%) | 1 (20%) |  |  |  |  |
| Appenzeller 2004 (n=9) | ECDS | 3 (33.3%) | 9 (100%) | 9 (100%) |  |  | 1 (11.1%) |  |  |  |  |
| Moko 2003 (n=10) | PRS | 5 (50%) | 1 (10%) |  |  |  |  |  |  |  |  |
| **Events (%)** | All subtypes (n=411) | 66 (16.1%) | 24 (5.8%) | 18 (4.4%) | 7 (1.7%) | 2 (0.5%) | 9 (2.2%) | 6 (1.5%) | 5 (1.2%) | 5 (1.2%) | 4 (1.0%) |
|  | ECDS (n=144) | 25 (21.6%) | 12 (10.3%) | 12 (10.3%) | 0 | 0 | 3 (1.9%) | 1 (0.6%) | 1 (0.6%) | 2 (1.2%) | 3 (1.9%) |
|  | PRS (n=86) | 13 (19.1%) | 8 (11.8%) | 0 | 0 | 0 | 0 | 3 (4.4%) | 3 (4.4%) | 0 | 0 |
|  | ECDS+PRS (n=84) | 21 (31.8%) | 3 (4.5%) | 4 (6.1%) | 0 | 1 (1.5%) | 5 (7.6%) | 2 (3%) | 1 (1.5%) | 3 (4.5%) | 0 |
| **Pooled prevalence %**  **(95% CI)** | All subtypes | 15.02 (6.95–27.28) | 3.99 (0.45–19.40) | 0.858 (0.001–12.35) | 0.024 (<0.001–1.205) | 0.271 (<0.001–1.473) | 1.009 (0.043–4.840) | 0.208 (<0.001–1.797) | 0.006 (<0.001–0.885) | 0.086 (<0.001–1.504) | 0.499 (<0.001–2.039) |
|  | ECDS | 19.754 (7.647–36.052) | 2.954 (0.005–56.930) | 3.043 (0.003–64.495) | 0.000 (0.000–2.549) | 0.000 (0.000–2.549) | 0.788 (<0.001–8.084) | 0.862 (<0.001–3.740) | 0.862 (<0.001–3.740) | 0.037 (<0.001–3.931) | 0.159 (<0.001–4.692) |
|  | PRS | 15.921 (1.548–47.707) | 11.765 (4.357–20.796) | 0.000 (0.000–4.310) | 0.000 (0.000–4.310) | 0.000 (0.000–4.310) | 0.000 (0.000–4.310) | 0.968 (<0.001–9.343) | 0.968 (<0.001–9.343) | 0.000 (0.000–4.310) | 0.000 (0.000–4.310) |
|  | ECDS+PRS | 27.138 (8.150–45.159) | 4.545 (0.528–11.494) | 9.091 (2.147–18.012) | 0.000 (0.000–4.438) | 0.958 (<0.001–6.463) | 4.619 (0.086–18.108) | 0.358 (<0.001–7.430) | 1.515 (<0.001–6.502) | 3.519 (0.016–11.269) | 0.000 (0.000–4.438) |

ECDS: *en coup de sabre*, PRS: Parry-Romberg syndrome.
